# Relationship between Adverse Childhood Experiences and Mental Health in Chinese Adolescents: Differences among Girls and Boys

**DOI:** 10.3390/children9050689

**Published:** 2022-05-09

**Authors:** Weiwei Jiang, Mingxia Ji, Xinli Chi, Xiaojiao Sun

**Affiliations:** 1 School of Psychology, Shenzhen University, Shenzhen 518060, China; jiangweiwei2020@email.szu.edu.cn (W.J.); xinlichi@szu.edu.cn (X.C.); 2 Center for Mental Health, Shenzhen University, Shenzhen 518060, China; 3 Guangming Branch of Shenzhen Institute of Educational Sciences, Shenzhen 518107, China; jkygmfy@szgm.gov.cn; 4 School of Physical Education and Sport Training, Shanghai University of Sport, Shanghai 200438, China

**Keywords:** adolescents, adverse childhood experience, depression symptoms, anxiety symptoms, sex differences

## Abstract

The negative effects of adverse childhood experiences (ACEs) on individual mental health have been widely demonstrated, yet fewer studies have examined the impact of ACEs on depression and anxiety of Chinese adolescents and their sex differences. This cross-sectional study surveyed 12421 adolescents aged 10–17 in Hechi City, Guangxi Province, to measure their levels of ACEs, depression symptoms, and anxiety symptoms. The results found that: (1) Girls were more likely to experience ACEs than boys (37.67% vs. 32.25%, χ^2^ = 39.97, *p* < 0.001). (2) Emotion-related ACEs were more likely to occur among girls, while physical maltreatment, violence, and family dysfunction related ACEs were more likely to occur among boys. (3) Adolescents with ACEs were more likely to develop depression (OR = 4.40) and anxiety symptoms (OR = 4.60) than those without ACEs; adolescents who have encountered “peer isolation” and “emotional neglect” are most likely to develop depression (OR = 6.09/5.04) and anxiety symptoms (OR = 6.14/4.94). (4) The dose-response relationship between the level of ACE exposure and the risk of depression/anxiety symptoms was significant (*p* < 0.05), i.e., the risk increased as ACE level increased. (5) Girls were more likely to develop depression and anxiety symptoms than boys with the same ACE level. This study deepens the understanding of the prevalence of ACEs, the effect of ACEs on depression and anxiety symptoms, and their sex differences among Chinese adolescents in the underdeveloped regions of China. It provides more empirical support for future work on adolescent mental health protection.

## 1. Introduction

According to Felitti et al. [1], Adverse Childhood Experiences (ACEs) are traumatizing events that occur in childhood (before 18), such as experiencing abuse or neglect, being involved in family conflicts or chaos, witnessing violence, etc., which may undermine individual’s sense of security, stability, and bonding. The initial study of ACEs, which was based on the health records of more than 17,000 American adults, found that ACEs (including three types of abuse and four types of family dysfunction in that study) were highly associated with health risk behaviors and diseases in adulthood [1]. Since then, many studies have reported high ACE prevalence in different populations [2,3,4]. Additionally, the negative effects of ACEs on physical and mental health have been widely documented [5,6,7,8,9]. However, most previous studies on ACEs were conducted in Western countries; such empirical research on Chinese adolescents is relatively lacking. It will be beneficial to conduct such studies among Chinese adolescents to provide more practical support for the future work of adolescent education and protection.

### 1.1. Prevalence of ACEs

The prevalence of ACEs (percentage of participants that experienced at least one type of ACE) varies widely in different studies due to the differences in sample populations, cultural backgrounds, measurement tools, etc. In most studies conducted in Western countries, the ACE prevalence rates were around or above 50%. For example, studies using data from the U.S. Behavioral Risk Factor Surveillance System reported that the ACE prevalence among U.S. adults was 61.55% between 2011 and 2014 and 60.9% between 2015 and 2017 [3,10]. The data from the U.S. National Survey of Children’s Health (2016) found that the ACE prevalence among U.S. adolescents (aged 12 to 17) was 55.7% [11]. Additionally, a national survey among adults in the U.K. reported an ACE prevalence of 46.4% [12]. Some studies in developing countries reported a higher prevalence of ACEs. For example, a study in Brazil reported that 85% of adolescents experienced at least one type of ACE [13]; another study found that the ACE prevalence among youth in South Africa was up to 90% [14]. So far, there have been few ACE studies in China, and most of them were based on a limited sample of college students. For instance, Wang and colleagues surveyed 990 college students and found that 43.82% of them had ever experienced ACEs [15]; another study involving 658 college students reported a prevalence of 44.8% [16]. The generalizability of the results was limited by the sample size and sampling method (recruited from one to two universities) in the above studies. Studies with randomized sampling and a large sample are required to discover the prevalence of ACEs among Chinese adolescents.

### 1.2. The Relationship between ACEs and Mental Health

The negative impact of ACEs on mental health has been widely documented. Numerous previous studies indicated that ACEs might increase the risk of anxiety, depression, addictive behaviors, PTSD, and even suicide [7,8,9,17,18]. Since the concept of ACEs encompasses multiple categories of negative experiences in childhood, some researchers believe it is necessary to discuss the differences in the effects of different categories and exposure levels of ACEs [7]. Some studies suggested that certain categories of ACEs may be associated with specific mental health problems [19] or that some ACEs may have stronger effects on mental health than other types of ACEs [20]. For example, a study based on U.S. children found that among the nine categories of ACEs, only “parental mental disorder” and “domestic violence” were associated with both depression and anxiety diagnosis; in contrast, only “physical abuse” and “parental incarceration” were not associated with both depression and anxiety symptoms [7]. It has also been argued that there is no significant difference in the impact of different categories of ACEs [21]. For instance, Elmore and Crouch [22] reported that all ACE categories included in their studies were significantly associated with children’s depression and anxiety symptoms. The findings for levels of ACEs exposure are more consistent, suggesting that higher levels of ACEs were associated with a greater risk of mental health problems [6,7]. Some studies reported a dose-response relationship between ACE levels and multiple mental health problems, such as depression, anxiety, substance addiction, suicidal ideation, etc. [23,24,25]. Although the relationship between categories or levels of ACEs and mental health has been wildly discussed in western countries, it remains unexamined among Chinese adolescents. 

### 1.3. Sex Differences in the Prevalence of ACEs and Their Effect on Mental Health

According to the theory of gender role orientation, individuals tend to believe that he or she possesses certain gender-typed characteristics consistent with the social culture. For example, boys are more likely to identify with a masculine or instrumental gender role, and girls are more likely to identify with a feminine or expressive gender role [26]. This identification process may also influence the prevalence and effects of ACEs. Studies have shown that sex/gender differences in mental health problems begin to emerge in adolescence and increase with age [26]. Therefore, adolescents may be the ideal subjects to explore sex/gender differences in the effects of ACEs.

Many researchers have examined sex/gender differences in ACE prevalence, but the findings are inconsistent. Most studies conducted in Western and Middle East countries have found a higher ACE prevalence in males [6,27,28,29]. Some studies from Korea and Brazil indicated that females are more likely to experience ACEs [13,30]. Additionally, some other studies in the U.S. reported that the difference in ACE prevalence between males and females was not significant [7,31]. Regarding the prevalence of individual categories of ACEs, most studies found that females were more likely to experience sexual assault [32,33], and males were more likely to experience other types of violence, such as physical abuse, domestic violence, and community violence [27,34]. Meanwhile, a study from Saudi Arabia reported a higher prevalence of sexual assault among men [28], while another study from Tunisia reported a higher prevalence of physical abuse among women [9]. In previous studies, findings of sex/gender differences in the level of ACE exposure were also inconsistent. Some studies from the U.S., Saudi Arabia, and Tunisia found that males were more likely to experience between four and five (or more) types of ACEs [9,28,35]. In contrast, some other studies from the U.S. and Brazil reported that females were more likely to experience between four and five (or more) types of ACEs [1,13,31].

Sex/gender differences in the effect of ACEs on mental health have also been widely discussed. Most studies concluded that females are more likely to develop depression, anxiety, or other mental health problems under equivalent ACE levels than males [27,28]. For example, Chapman and colleagues found that compared to men, women who experienced five or more ACEs have a higher risk of depression [24]; El and colleagues noted that experiencing either type of ACEs increased the risk of addictive behaviors in women, but not in men [9]. In some specific studies, however, it has also been found that males may be more affected by ACEs than females. Such as a longitudinal study among U.S. students reported that childhood sexual assault was more strongly associated with depression symptoms and addictive behaviors in boys than in girls [36]. There are also different opinions that the impacts of ACEs are more consistent than differential across sexes or that differences exist only under certain conditions [37]. For example, some studies conducted among U.S. adults found no significant sex/gender differences in the effects of ACEs on depression symptoms, substance abuse, and suicide risk [23,35]. Meanwhile, Dunn and colleagues’ study among adolescents found that such sex/gender differences were significant only at a low level of ACE, with sex/gender differences decreasing to nonsignificant as the level of ACE increased [20]. Results about the sex/gender differences of ACEs’ effects on mental health were conflicting in different populations and cultural backgrounds, and such research among Chinese adolescents is still lacking. 

### 1.4. Current Research

Despite the large amount of research on the prevalence, effects, and sex differences of ACEs in Western countries, such studies are comparatively lacking in the Chinese population. Additionally, most of the previous ACE studies were conducted with adults (over 18). Since the measurement of ACEs relies on retrospective self-reports, Hunt et al. [7] suggested that taking adults in ACE studies is prone to memory bias and may ignore the proximal effect of ACEs. In addition, a longitudinal study pointed to a stronger association between ACEs and mental health in adolescence than in adulthood [38]. In conclusion, it is necessary to conduct a large sample ACE study to explore the prevalence, effects, and sex differences of ACEs among Chinese adolescents. Meanwhile, given that low socioeconomic status is one of the risk factors for ACEs [39], the present study considers it more relevant to select adolescents from underdeveloped regions of China. Nowadays, depression and anxiety are the most common concern for adolescent mental health in Chinese society. Since many researchers have pointed out that ACEs are significant risk factors for depression and anxiety in adolescents [40,41], this study chooses the symptoms of depression and anxiety as outcome variables. This study raises the following two research questions: (1) the prevalence of ACEs in adolescents and their sex differences; (2) the effects of individual categories and exposure levels of ACEs on depression and anxiety symptoms in adolescents and their sex differences.

## 2. Method

### 2.1. Participants

Data of this study were collected in May 2020 in the Yizhou District, Hechi City, Guangxi Province of southern China, an underdeveloped area with about 20.15% of its population living below the poverty line in 2015 [42]. A stratified random sampling method was used to select 12 primary schools and 17 middle and high schools out of the total 161 public schools in this district. Online questionnaires were sent to all students from grades 4 to 11 in the selected schools, and a total of 14,392 responses were received. Those responses with participants younger than 10, completion time less than 300 s, and answers with apparent regularity were excluded as invalid responses. After eliminating, a total of 12,421 valid responses were retained.

### 2.2. Procedure

The process of data collection was assisted by the local education bureau and permitted by school administrations. Participants and parents/guardians were informed about the study prior to data collection by class teachers. It has been explained that all data would be aggregated before being analyzed, and all personal information would be kept strictly confidential. Participation in the study was voluntary. A consent form was electronically signed by both participants and their guardians/parents before completing the questionnaire. Standardized instructions were contained in the questionnaire. Since all items related to critical variables in this study were set as forced choices, missing data were automatically excluded. Recruitment and data collection procedures were approved by the Human Research Ethics Committee (No. 2020005) of Shenzhen University. 

### 2.3. Measurement

ACEs were measured using the Chinese version of the revised Adverse Childhood Experiences Questionnaire (ACEQ-R), which was revised based on adolescent participants aged 10 to 17 [43]. The original ACEs questionnaire assesses ten aspects of ACEs: parental emotional abuse, parental physical abuse, sexual assault, parental emotional neglect, parental physical neglect, witnessing domestic violence, family member drug/alcohol problems, family member mental illness, parental separation/divorce, and family member incarceration. Additionally, the four more items added in the revised version are peer victimization, peer isolation/rejection, exposure to community violence, and low socioeconomic status. Responses to each item were either “yes” or “no.” Responding “yes” will be coded as “1” and be considered to have corresponding ACEs; responding “no” will be coded as “0.” Scores for ACEQ-R were calculated based on the 14 items and range from 0 to 14. The score of ACEQ-R represents the number of ACEs participants have experienced, which is also considered as the levels of ACE exposures. This scale was first translated into Chinese and then back-translated. Three bilingual experts were invited to review the translation, followed by a pre-test involving 30 adolescents to determine the readability. This scale demonstrated high internal reliability (Cronbach α = 0.87) and good fit indices (χ^2^ = 4362.84, df = 75, *p* < 0.001, CFI = 0.92, TLI = 0.90, RMSEA = 0.07, SRMR = 0.04) in confirmatory factor analysis in this study.

Depression symptoms were measured using the Chinese version of the Patient Health Questionnaire (PHQ-9), which was initially developed by Spitzer et al. [44], translated and validated by Bian et al. [45]. The Chinese version of the PHQ-9 has been widely used and validated in Chinese adolescents [46]. This scale consists of 9 items that assessed the level of depression symptoms over the past two weeks (e.g., feeling down, depressed, or hopeless). Each item is rated on a Likert scale of 1 to 4 (0 = “Not at all”, 3 = “Nearly every day”). The total score of the 9 items indicates the level of depression, with higher scores indicating a higher level of depression. Based on the suggestion of the cutoff score in previous studies [47], participants with a PHQ-9 score of 10 and above were considered to have depression symptoms in this study. The Cronbach’s α coefficient of the scale in this study was 0.90.

Anxiety symptoms were measured using the Chinese version of the Generalized Anxiety Disorder Scale (GAD-7), which was initially developed by Spitzer et al. [48], translated into Chinese and validated among Chinese patients by He and colleagues [49]. The Chinese version has been proved with good reliability and validity among adolescents [50]. This scale consists of 7 items that assessed the level of generalized anxiety symptoms over the past two weeks (e.g., feeling nervous, anxious, or on edge). Each item is rated on a Likert scale of 1 to 4 (0 = “Not at all”, 3 = “Nearly every day”). The total score of the 7 items indicates the level of anxiety, with higher scores indicating a higher level of anxiety. Based on the suggestion of the cutoff score in previous studies [51], participants with a GAD-7 score of 10 and above were considered to have anxiety symptoms in this study. The Cronbach’s α coefficient of the scale in this study was 0.92.

### 2.4. Statistical Analysis

SPSS (version 26) was used for statistical analysis in this study. According to previous studies, the prevalence of ACEs was calculated as the percentage of participants who have experienced at least one type of ACE (ACEQ-R total score ≥ 1); the prevalence of individual categories of ACEs was calculated as the percentage of participants who answered “yes” to the specific item of ACEQ-R [43]. The level of ACE exposure was according to the total score of ACEQ-R. Participants with ACEQ-R ≥ 4 were combined into one class and valued as 4. Thus, the level of ACE was converted into a categorical variable of 0 to 4 points [7,28]. Firstly, descriptive statistics were conducted for the prevalence of ACEs, depression symptoms, anxiety symptoms, and demographic variables. Next, adjusted logistic regression was used to analyze the associations between sex and the occurrence of ACEs/depression/anxiety, as well as the association between the category/level of ACE and the occurrence of depression/anxiety. The occurrence of ACEs, depression and anxiety were treated as binary variables in the equation. Demographic variables (e.g., age, father’s/mother’s education, who the respondent lives with) were included as control variables. At last, the linear trend chi-square test was used to examine the dose-response relationship between levels of ACE exposure and the risk of depression/anxiety. 

## 3. Results

### 3.1. Descriptive Characteristics of the Participants

The final sample in this study consisted of 12,421 adolescents aged 10 to 17 (M = 13.48, SD = 1.47), of which girls accounted for 52.47%, and boys accounted for 47.53%. More detailed descriptive characteristics of the participants are shown in Table 1.

### 3.2. Prevalence of ACEs and Depression/Anxiety Symptoms in Adolescents and Their Sex Differences

The results for ACE prevalence (Table 2) showed that 35.09% of participants in this study had experienced at least one type of ACE; more girls have experienced ACEs than boys (37.67% vs. 32.25%, χ^2^ = 39.97, *p* < 0.001). In terms of the individual categories of ACEs, the three categories with the highest prevalence were peer isolation (21.28%), emotional abuse (20.34%), and emotional neglect (14.81%); the three categories with the lowest prevalence were family member drug/alcohol problems (3.55%), family member mental illness (3.87%), and sexual assault (4.11%). ACEs related to emotions (e.g., peer isolation, emotional neglect, and emotional abuse) were more likely to occur in girls. In contrast, ACEs related to physical maltreatment (e.g., sexual assault, physical abuse, physical neglect), violence (e.g., witnessing community violence, peer victimization), and family dysfunction (e.g., family member drug/alcohol problems, family member incarceration) were more likely to occur in boys. Regarding the level of ACE exposure, 17.39% of the participants experienced one type of ACE, and 5.21% experienced four or more types of ACE. Girls were more likely to experience one to three types of ACEs, while boys were more likely to experience four or more types of ACE (5.79% vs. 4.51%, χ^2^ = 10. 47, *p* < 0.01).

The prevalence results of depression and anxiety symptoms are shown in Table 2. The prevalence of depression symptoms was 17.85% in this study, which was higher among girls than among boys (22.07% vs. 13.19%, χ^2^ = 166.25, *p* < 0.001). The prevalence of anxiety symptoms was 10.26% in this study, which was higher among girls than among boys (13.15% vs. 7.06%, χ^2^ = 124.70, *p* < 0.001).

### 3.3. The Association between ACEs and Depression/Anxiety Symptoms and Their Sex Differences

The logistic regression results estimating the association between individual ACE categories and depression/anxiety symptoms are demonstrated in Table 3. Each category of ACEs significantly increased the risk of depression (OR range 1.54 to 6.78) and anxiety symptoms (OR range 1.75 to 6.85) in boys and girls. The strength of effects of individual ACE categories on depression and anxiety symptoms tends to be consistent across sexes. For example, “peer isolation” had the strongest association with depression (OR = 5.26/6.78) and anxiety symptoms (OR = 5.27/6.85) in both boys and girls, followed by “emotional neglect” (depression OR = 4.96/5.26, anxiety OR = 5.11/5.00). In contrast, “low socioeconomic status” had the weakest association with depression (OR = 2.07/1.54) and anxiety symptoms (OR = 2.55/1.75) in both boys and girls.

The logistic regression results estimating the association between the level of ACE exposure and depression/anxiety symptoms are displayed in Table 4. Even the lowest level of ACE exposure had a significant effect on the occurrence of depression and anxiety symptoms. The odds ratio increased as ACE level increased. A significant dose-response relationship (*p* < 0.05) was observed between ACE level and depression/anxiety symptoms in the full sample, boys, and girls. With each level increasing in ACE exposure, the risk of depression/anxiety symptoms increased 2.5 to 3.6 times. In terms of the sex differences, the effect of ACEs on depression/anxiety symptoms was higher for girls than boys at all levels of ACEs. For example, the odds of depression and anxiety symptoms increased 7.52 and 7.99 times, respectively, for boys with four or more ACEs compared to those without ACEs, while the odds increased 8.49 and 10.08 times for girls with four or more ACEs compared to those without ACEs.

## 4. Discussion

### 4.1. ACE Prevalence in Adolescents and Sex Differences

In this study, 35.09% of the adolescents have experienced at least one type of ACE, indicating that ACEs are common among Chinese adolescents, and require more attentions from educators and researchers. However, this prevalence is at a comparatively low level relative to the ACE prevalence (above 50%) reported by previous studies in many other countries [1,2,3,4]. Similar to this result, some studies in the U.S. found that the prevalence of ACEs in Asian Americans is significantly lower than in other ethnicities [6,11]. A possible explanation is that social culture may influence adolescents’ perceptions of maltreatment. Asian societies are considered to be more accepting of strict parenting styles and punishments than western societies [52,53]. Growing up in this culture, some adolescents who have been punished or reprimanded may not perceive it as a sort of “abuse.” In addition, Chinese culture values interpersonal harmony, and some researchers considered that adolescents in this culture might reduce reporting conflicts and adversities to avoid social stigma [6]. On the other hand, the low prevalence of some specific ACE categories may reflect the current status of China society. For example, the prevalence of “family drug/alcohol problem” was 3.55% in this study, lower than those reported in similar studies in the United States (9.2~15.1%) [7,43], which is consistent with the status of the overall drug/alcohol problem in China. According to China Drug Report 2020, there were about 1.8 million (about 1.3‰ of the population) illicit drug users in China in 2020 [54]. Additionally, a systematic review covering 38 studies with 1,304,354 individuals reported that the lifetime and current prevalence of alcohol dependence in China was 1.4% and 1.5% [55]. In comparison, the 2020 National Survey on Drug Use and Health of the United States reported that, among U.S. people aged 12 or older, 21.4% (or 59.3 million people) used illicit drugs in 2020, and 22.2% (or 61.6 million people) had binged alcohol in the past month [56]. Therefore, both subjective and objective factors may have contributed to the low ACE prevalence in this study. 

In this study, girls reported a higher general ACE prevalence than boys (37.67% vs. 32.25%). Specifically, girls were more likely to experience peer isolation (26.75% vs. 15.24%), emotional abuse (21.11% vs. 19.50%), and emotional neglect (18.29% vs. 10.96%). This result may be explained by the adolescents’ development characteristics and sociocultural factors. Girls usually become more emotionally fragile, overthinking, and eager for attention during adolescence [57]. These characteristics may make it easier for girls to feel isolated by their peers and become victims of psychological violence in schools. In addition, some social studies found that the son preference is still common in rural areas of Guangxi Province, and girls usually take on more household chores and enjoy fewer family resources than boys [58,59]. Thus, girls may encounter more emotional neglect and abuse in their families. Boys, however, reported more physical maltreatment and violence-related ACEs than girls in this study, which is consistent with some previous studies [9,32]. The high prevalence of physical abuse and neglect reported by boys might be partly caused by parental corporal punishment. As mentioned above, Chinese parents are usually strict with their children. Meanwhile, they may expect more from boys in a patriarchal tradition, so boys may be disciplined more harshly than girls and be more likely to get corporal punishment from their parents [53]. This result can also be explained by the gender role socialization process, which declares that boys may express more disharmonious emotions (e.g., anger) through externalizing behaviors [60] and get themselves into more physical conflicts or even violent ACEs. 

### 4.2. Effects of ACEs on Adolescents’ Depression and Anxiety Symptoms and Their Sex Differences

This study indicated that exposure to any category or level of ACE could significantly increase the risk of depression and anxiety symptoms for adolescents. Additionally, the risk increased with the increase in ACE level. The results were consistent with the findings of many previous studies [7,22,35]. The learned helplessness theory suggests that repeated uncontrollable adverse events cause the feeling of helplessness. It may lead to depression or anxiety symptoms if the feeling of helplessness becomes a habit and is generalized into ordinary life situations [61,62]. The results can also be explained by the allostatic load model from a neurophysiological perspective. In the nervous system, adversities may lead to structural and functional abnormalities in stress-sensitive brain regions and further influence adolescent brain maturation. In the endocrine system, the chronic stress caused by adversities may overstimulate the HPA (hypothalamic–pituitary–adrenal) axis, thus impairing one’s emotional regulation ability. Such a process may ultimately cause an allostatic overload and enhance the risk of depression and anxiety symptoms in adolescents [63,64].

This study further found that adolescents who experienced “peer isolation” and “emotional neglect” had the highest risk of developing depression and anxiety symptoms in both boys and girls, which is a finding that few studies have reported before. The developmental characteristics of the adolescents may be one of the factors in response to this result. Adolescents tend to be egocentric [65], overthinking, and eager for attention [57]. These characteristics may contribute to their sensibility and vulnerability to the feeling of “isolation” and “neglect.” In addition, studies have pointed out that adolescents tend to reduce their emotional dependence on parents and look for more connections and support from their friends [66]. Therefore, it is not difficult to understand that “peer isolation” had the most substantial impact on adolescent depression and anxiety symptoms among all ACE categories.

Regarding sex differences, girls were more susceptible to depression and anxiety symptoms from ACEs, which is consistent with some previous studies [28]. Some researchers explained this result with the theory of gender role orientation. Influenced by the orientation process of gender roles, boys are more likely to express distress through external behaviors, while girls are more likely to turn their distress inward and exhibit symptoms of depression and anxiety [26]. Some other researchers explained this difference from the perspective of attribution style. It is suggested that boys use more external attribution (e.g., blaming others) in adverse events; in contrast, girls use more internal attribution (e.g., self-blame) [67,68,69] and may further develop into internalizing problems under adversities [34]. In addition, some researchers pointed out that the social standards are stricter for girls in traditional Chinese culture [70]. This may exacerbate girls’ self-blame and eventually lead to mental health problems, such as depression and anxiety.

### 4.3. Limitations and Directions for Future Research

Firstly, data of this study were collected from one district of Hechi City, which may result in sample homogeneity to some degree. The findings of the study, especially the ACE prevalence, may not be able to be generalized to other regions of China. A nationwide sampling needs to be considered in further studies. Secondly, although the age ranged from 10 to 17 in this study, 70% of the participants were aged between 13 to 15. This age imbalance may cause some bias in results and reflect more characteristics of middle adolescence instead of integrated adolescence. A balanced sample size for each age group needs to be considered in future studies. Thirdly, the data were collected after the first outbreaking of COVID-19. Although there were few COVID-19 cases in Guangxi Province by that time, the tension caused by lockdown may have influenced the mental health results or caused recollection bias. Future studies should minimize the influence from environmental factors, or may consider conducting the study after the pandemic. Finally, this study used cross-sectional data, which cannot reflect the causal association between ACEs and psychological outcomes. Further longitudinal studies are required to better understand the impact of ACEs on mental health. 

## 5. Conclusions

(1) This study found the prevalence of ACEs in the adolescent participants was 35.09%. (2) There were significant sex differences in ACE prevalence: girls were more likely to experience ACEs than boys; boys were more likely to experience four or more ACEs; emotion-related ACEs were more likely to occur in girls, while physical maltreatment, violence, and family dysfunction-related ACEs were more likely to occur in boys. (3) Any category of ACE significantly increased the risk of depression and anxiety symptoms among both boys and girls; “peer isolation” and “emotional neglect” showed the most substantial influence on depression and anxiety symptoms for both boys and girls. (4) Any level of ACE significantly increased the risk of depression and anxiety symptoms among both boys and girls; a significant dose-response relationship was observed between the ACE level and depression/anxiety symptoms, meaning the risk increased with the increase in ACE level. (5) Girls were more likely to develop depression and anxiety symptoms than boys with the same ACE level.

This study deepened our understanding of ACE prevalence, the influence of ACEs on mental health, and their sex differences among adolescents from underdeveloped regions of China. First, we found that ACEs are prevalent among adolescents, and any category or level of ACE may have profound negative effects on adolescents’ mental health. It indicates that parents, teachers, and other relevant persons should be broadly educated about ACEs and their harm to prevent any type of ACE. Second, the most common and influential ACEs that adolescents reported were peer isolation, emotional abuse, and neglect, rather than violence or physical abuse, as traditionally believed. It reminds the importance of adolescents’ peer relationships, and some actions could be taken in schools, such as activities that foster friendships, training to improve adolescents’ interpersonal skills, or psychological support for students who may be isolated. Meanwhile, to reduce emotional neglect and abuse in families, it is necessary to conduct more parental supporting programs that improve parenting style, family harmony, and parent-child relationships. Finally, we found that girls are more likely to experience ACEs and are more affected by them, especially emotion-related ACEs. It suggests that the protection for girls should be further strengthened, and the idea of equality between girls and boys should be further promoted, especially in underdeveloped areas of China. More specifically, in the protection for girls, responding to their emotional needs is particularly important.

## Figures and Tables

**Table 1:** Descriptive Characteristics of the Participants.

Variables		No.	%
Sex	boy	5904	47.53
	girl	6517	52.47
Age	10–12	3062	24.65
	13–15	8579	69.07
	16–17	780	6.28
Father education	junior middle school and below	8802	70.86
	high school and junior college	2502	20.14
	college and above	1117	8.99
Mother education	junior middle school and below	9102	73.28
	high school and junior college	2213	17.82
	college and above	1106	8.90
Lives with	parents	7145	57.52
	signal parent	2255	18.15
	no parent	3021	24.32

**Table 2:** Prevalence of ACEs, depression symptoms, and anxiety symptoms.

Variables	Full Sample (%)	Boy (%)	Girl (%)	χ^2^
Occurrence of ACEs	35.09	32.25	37.67	39.97 ***
Category of ACEs				
Emotional abuse	20.34	19.50	21.11	5.01 *
Physical abuse	7.56	9.40	5.89	54.55 ***
Sexual assault	4.11	5.15	3.16	31.10 ***
Emotional neglect	14.81	10.96	18.29	132.02 ***
Physical neglect	4.12	4.89	3.42	17.01 ***
Parents’ divorce/separation	10.07	9.96	10.17	0.16
Witnessing domestic violence	4.62	4.96	4.31	2.98
Family drug/alcohol problem	3.55	4.51	2.69	29.97 ***
Family mental illness	3.87	4.03	3.73	0.76
Family member incarceration	5.61	6.15	5.13	6.12 *
High peer victimization	5.45	6.00	4.96	6.50 *
High peer social isolation	21.28	15.24	26.75	244.63 ***
Witnessing community violence	4.40	5.49	3.41	31.93 ***
Low socioeconomic status	8.69	8.71	8.68	0.00
Level of ACE exposure				
1 type of ACE	17.39	15.55	19.06	26.55 ***
2 types of ACEs	8.25	7.05	9.34	21.62 ***
3 types of ACEs	4.33	3.86	4.76	6.00 *
4+ types of ACEs	5.12	5.79	4.51	10.47 **
Mental health problems				
Depression symptoms	17.85	13.19	22.07	166.25 ***
Anxiety symptoms	10.26	7.06	13.15	124.70 ***

Note: * *p* < 0.05, ** *p* < 0.01, *** *p* < 0.001.

**Table 3:** Logistic regression estimating the association between ACE categories and depression/anxiety symptoms.

Category of ACEs	Depression Symptoms (OR)			Anxiety Symptoms (OR)		
	Full Sample	Boy	Girl	Full Sample	Boy	Girl
Emotional abuse	3.56	3.25	3.86	3.44	3.37	3.58
Physical abuse	2.70	2.95	2.56	2.77	3.00	2.65
Sexual assault	2.54	2.65	2.38	2.72	2.58	2.82
Emotional neglect	5.04	4.96	5.26	4.94	5.11	5.00
Physical neglect	3.16	3.21	3.08	3.68	3.64	3.71
Parents’ divorce/separation	2.01	2.34	1.87	2.14	2.61	1.97
Witnessing domestic violence	3.05	3.34	2.65	3.29	3.29	3.21
Family drug/alcohol problem	2.80	3.04	2.30	2.78	3.07	2.41
Family mental illness	3.27	2.30	3.50	3.54	3.32	3.74
Family member incarceration	2.17	2.63	1.79	2.23	2.26	2.20
High peer victimization	3.69	3.57	3.87	4.04	3.89	3.85
High peer social isolation	6.09	5.26	6.78	6.14	5.27	6.85
Witnessing community violence	3.37	4.13	2.50	3.52	3.76	3.26
Low socioeconomic status	1.69	2.07	1.54	1.98	2.55	1.75

Note: Participants without the corresponding category of ACEs were used as the reference group in each category; all demographic variables were used as control variables in the full sample; demographic variables except sex were used as control variables in the boy/girl sample; all OR values were significant with *p* < 0.001.

**Table 4:** Logistic regression estimating the association between level of ACE exposure and depression/anxiety symptoms.

ACEs Exposure	Depression Symptoms (OR)		Anxiety Symptoms (OR)	
	0 ACEs as the Reference Group	Previous Level as the Reference Group	0 Aces as the Reference Group	Previous Level as the Reference Group
Full sample				
0 ACE	1.00	1.00	1.00	1.00
1 ACE	2.69	2.73	2.73	2.76
2 ACEs	5.46	3.38	5.54	3.40
3 ACEs	6.91	2.91	6.26	2.59
4+ ACEs	8.08	2.55	9.18	2.96
Boys				
0 ACE	1.00	1.00	1.00	1.00
1 ACE	2.45	2.48	2.52	2.54
2 ACEs	4.64	3.01	4.77	3.05
3 ACEs	6.03	2.72	5.12	2.26
4+ ACEs	7.52	2.62	7.99	2.86
Girls				
0 ACE	1.00	1.00	1.00	1.00
1 ACE	2.84	2.88	2.87	2.90
2 ACEs	6.00	3.61	5.99	3.59
3 ACEs	7.55	3.05	6.97	2.79
4+ ACEs	8.49	2.52	10.08	3.06

Note: Participants without the corresponding category of ACEs were used as the reference group in each category; all demographic variables were used as control variables in the full sample; demographic variables except sex were used as control variables in the boy/girl sample; all OR values were significant with *p* < 0.001.

## Data Availability

The datasets generated during and/or analyzed during the current study are available from the corresponding author on reasonable request.
